# Formation Energies of Antiphase Boundaries in GaAs and GaP: An *ab Initio* Study

**DOI:** 10.3390/ijms10125104

**Published:** 2009-11-25

**Authors:** Oleg Rubel, Sergei D. Baranovskii

**Affiliations:** 1 Thunder Bay Regional Research Institute, Thunder Bay, 290 Munro St, P7A 7T1, Canada; 2 Department of Physics and Material Sciences Center, Philipps-University Marburg, Marburg 35032, Germany; E-Mail: baranovs@staff.uni-marburg.de (S.D.B.)

**Keywords:** antiphase boundary, III-V semiconductors, formation energy, density-functional theory

## Abstract

Electronic and structural properties of antiphase boundaries in group III-V semiconductor compounds have been receiving increased attention due to the potential to integration of optically-active III-V heterostructures on silicon or germanium substrates. The formation energies of {110}, {111}, {112}, and {113} antiphase boundaries in GaAs and GaP were studied theoretically using a full-potential linearized augmented plane-wave density-functional approach. Results of the study reveal that the stoichiometric {110} boundaries are the most energetically favorable in both compounds. The specific formation energy γ of the remaining antiphase boundaries increases in the order of γ_{113}_ ≈ γ_{112}_ < γ_{111}_, which suggests {113} and {112} as possible planes for faceting and annihilation of antiphase boundaries in GaAs and GaP.

## 1. Introduction

**Figure 1.**
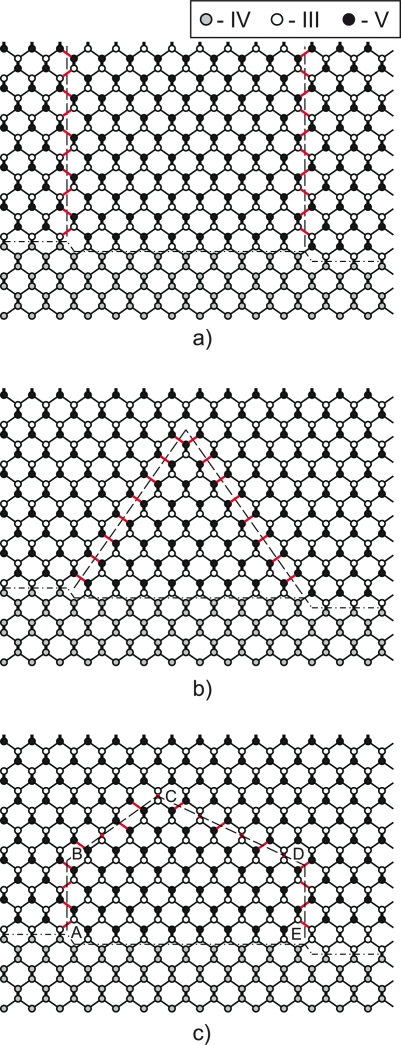
(color online) Formation of the APB’s at the interface between group IV and group III-V semiconductors. ABP’s emerge at monosteps on the group IV (001) surface. Several possibilities are considered: (a) APB growth along the {110} plane extended to the free surface, (b) APB growth along the {111} plane and subsequent annihilation, (c) APB growth along the {110} plane (segments AB and DE), subsequent kinking in {112} plane (BC) or {113} plane (CD) and annihilation. The wrong bonds at the interface between inversion domains are marked red.

Epitaxial junctions of III–V/IV semiconductors that are closely matched in lattice spacing are desirable for various technological applications including GaAs/Ge monolithic tandem solar cells [1] and the integration of optically active III–V semiconductors into the silicon-based technology [2, 3]. In spite of advancement in the growth technology of III–V/IV semiconductor epilayers [4–6], the fabrication of high-quality structures remains a challenge, so far. The major problem is caused by the interface between non-polar (group IV) and polar (group III–V) semiconductors. As illustrated in Figure 1, the presence of monoatomic steps on the (001) surface of a group-IV semiconductor leads to the formation of antiphase boundaries (APB’s) in an overgrown layer of the III–V semiconductor. APB’s can either kink and self-annihilate [7] or continue growing along {110} plane across the deposited layer as shown in Figure 1. The latter case leads to the structure degradation and is highly unfavorable.

Previous studies of GaAs/Ge and GaP/Si epitaxial layers report the predominant formation of {110} APB’s [8–11] along with their faceting in other planes [9]. A recent TEM investigation [12] of GaP layers heteroepitaxially grown on exact (001) Si suggest that both scenarios described above are possible depending on the growth conditions. At relatively low growth temperatures, {110} APBs tend to form, while higher growth temperatures promote APB kinking and annihilation [12]. The faceting phenomena observed at APB’s indicate that the energy associated with the presence of APB has a correlation with the boundary plane [9]. Unfortunately, there is no explicit experimental evidences of what is a preferred plane for the annihilation of APB’s in GaAs and GaP. For example, various experimental studies [10, 12,13] suggest {111}, {331}, and {113} planes for annihilation of APB’s in GaP/Si. This indicates that the APB formation energy is only one of many factors which determine favorable conditions for the APB annihilation.

Several methods have been proposed to account for the energetics of APB formation. Petroff [14] estimated the specific APB formation energy γ based on the the wrong bond density and the energy of the wrong bond *E*_wb_ calculated for antisite defects. Assuming *E*_wb_ = 0.3 eV for the case of GaAs [15], simple bond counting arguments yield γ = 27 and 22 meV/Å^2^ for the {110} and {111} APB’s, respectively. In this case, the difference between the specific formation energies of various APB’s is solely governed by the difference in the wrong bond density. The calculations reveal that the formation energy of the {111} APB’s is lower than the {110} APB’s. This result contradicts the first-principle calculations [16] that suggest the opposite trend, hereby indicating that the simple bond counting model is not appropriate for calculating the energetics of the APB’s.

Dandrea *et al*. [17] proposed a more elaborate model for calculating the formation enthalpies in heterovalent superlattices, which was later adopted and extended by Vanderbilt and Lee [16] and Lambrecht *et al*. [18] to APB’s in heterovalent semiconductors. In addition to the wrong bond contribution to the APB formation energy, this model includes effects associated with the charge transfer that takes place between III-III and V-V bonds. Since a III-III bond has a deficiency of $\frac{1}{2}$ electron and a V-V bond has an excess of $\frac{1}{2}$ electron, they are expected to behave as acceptor and donor states, respectively, thereby giving rise to an uncompensated system of fractionally occupied localized states [17]. Charge transfer from the donor V-V bond to the acceptor III-III bond lowers the APB formation energy by a value proportional to the magnitude of compensation. In addition, the charge transfer leads to a lattice of charges giving rise to the electrostatic (Madelung) contribution to the APB formation energy. The amount of compensation depends on the electrostatic energy of the ensuing arrangement of charged states [17]. In the case of stoichiometric APB, for example the {110} APB that has an equal number of III-III and V-V bonds in the APB plane, complete compensation is expected due to the *intra*-plane charge transfer, which results in a lower formation energy. Conversely, compensation in non-stoichiometric APB’s, such as {111}, involves the *inter*-plane charge transfer, which produces a potential drop across the antiphase domain. The potential drop hinders the charge transfer for distant non-stoichiometric APB’s, since the magnitude of the potential drop is limited by the band gap [16–18], which leads to a higher formation energy. The latter result agrees with the first-principle calculations [16].

From the arguments presented above, it is evident that, though being geometrically feasible [7], APB kinking and annihilation in the {111} plane is energetically highly unfavorable. The question arises as to whether there is an alternative to the {111} plane for APB annihilation. Here we report one possible scenario of APB annihilation that involves kinking in the {112} or {113} plane (Figure 1c). In contrast to the {111} APB, the {113} APB is partly non-stoichiometric, and a partial intra-plane compensation is expected; the {112} APB is stoichiometric, and full compensation due to the intra-plane charge transfer can be achieved. Therefore, the formation energy of the {112} and {113} ABP’s should be lower than that for the {111} APB. The purpose of this study is to verify this assumption by performing density-functional theory (DFT) calculations of the {110}, {111}, {112}, and {113} APB formation energies in GaAs and GaP. In addition to the previous theoretical study [16], we (i) consider {112} and {113} APB’s, (ii) report data for GaP, (iii) include relaxation of atomic positions into total energy calculations, and (iv) extend calculations to distant APB’s that eliminates the necessity of extrapolation of the formation energy and avoids the corresponding error.

## 2. Computational Details

**Figure 2.**
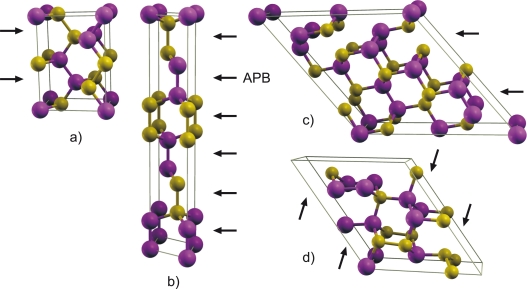
(color online) Atomic structure of the smallest unit cells representing various APB’s: (a) {110}, (b) {111}, (c) {113}, and (d) {112}. Arrows indicate the APB planes. (Visualized with XCrySDen [22].)

The specific formation energy of various APB’s is calculated using a supercell approach and defined as

(1) $$\gamma \;=\;\frac{E_{\text{tot}}^{\text{APB}}\;-\;E_{\text{tot}}^{\text{bulk}}}{\Omega }$$

where $E_{\text{tot}}^{\text{APB}}$ is the total energy of a supercell that contains APB, $E_{\text{tot}}^{\text{bulk}}$ is the total energy of an identical supercell representing an ideal bulk crystal with the same number of atoms and symmetry, and Ω is the corresponding APB cross-sectional area. Examples of the APB supercell structures are shown in Figure 2. Since all supercells are stoichiometric (*i.e.*, contain equivalent number of group-III and group-V atomic species), there is no need to introduce chemical potentials into Equation (1).

It is worth noting that the structural models of APB’s investigated here are viewed in DFT under the periodical boundary conditions. Each structure contains an even number of equidistant APB’s running in parallel planes. As a consequence, it is impossible to create a structure where one type of bonds, cationcation or anion-anion, will dominate over another one. It is also impossible to separate the wrong bond energy to that associated with cationcation and anion-anion bonds for the same reason. Therefore, all energetic characteristics calculated here are average by nature.

Total energy calculations were carried out in the framework of the full-potential linearized augmented plane wave method using Wien2k DFT package [19]. The volume of supercells was partitioned onto an interstitial region and non-overlapping spheres with radii of 2 bohr centered at the nucleus of the individual atoms. The product of the atomic sphere radius and of the plane wave cut-off in *k*-space (the so-called *RK*_max_ parameter) was equal to 7 for all structures, which corresponds to the cut-off energy of approximately 12 Ry. The local density approximation [20] was employed for the exchange-correlation functional. The energy needed to separate core and valence electrons was set to −6 Ry, wich results in treating of semi-core electrons as valence electrons.

The Brillouin zone of the supercells was sampled using a shifted Monkhorst-Pack [21] *k*-point mesh. The mesh density was adjusted for each individual structure based on the convergence of the calculated APB formation energy. For APB’s with a large interface separation that posses metallic electronic structure, the mesh density was as high as 120 k-points in the irreducible Brillouin zone of a 48-atom supercell. Temperature broadening scheme with an energy parameter equal to 0.005 Ry was applied to enhance the convergence when calculating the Fermi energy and weights of each band state.

For all structures optimization of internal degrees of freedom was performed. The structural optimization was continued until the forces acting on the atoms were below 2 mRy/bohr. Accordingly, the atomic positions, the APB formation energies, and the energies of wrong bonds were determined with the accuracy of 0.005 Å, 1 – 2 meV/Å^2^, and 0.01 eV, respectively.

## 3. Results and Discussion

**Figure 3.**
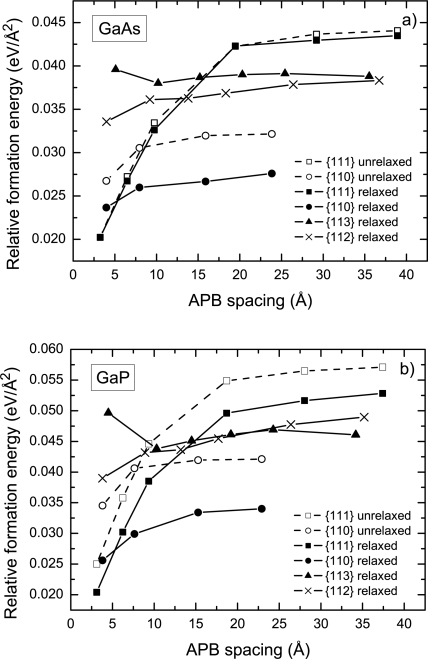
Specific formation energy of the {110}, {111}, {112}, and {113} APB’s in GaAs (a) and GaP (b). ”Relaxed” and ”unrelaxed” refer to calculations that include or exclude relaxation of atomic coordinates within the supercell.

Initially, the results of the calculations were verified through a comparison with the results of Vanderbilt and Lee [16] for the specific formation energy of the {110} and {111} APB’s in GaAs calculated using pseudopotential DFT. Since Vanderbilt and Lee [16] did not include the optimization of internal degrees of freedom (position of atoms) in their calculations, this factor was temporary omitted in order to eliminate a possible source of discrepancy. The results of the calculations for the specific formation energy of the {110} and {111} APB’s in GaAs as a function of APB spacing are shown in Figure 3(a) with dashed lines corresponding to the unrelaxed structures. Both the {110} and {111} APB’s feature an increase in formation energy with increasing APB spacing, until saturation occurs at the value of $\gamma_{\left\{110\right\}}^{\text{GaAs}}\;=\;32$ and $\gamma_{\left\{111\right\}}^{\text{GaAs}}\;=\;44$ meV/Å^2^. The calculated asymptotic formation energies are in good agreement with the corresponding values of 34 and 44 meV/Å^2^ obtained in Ref. [16] using an extrapolation technique for distant {110} and {111} APB’s, respectively.

Subsequently, a study of the effect of the relaxation of atomic positions on APB formation energies was conducted. The study revealed that the relaxation of atomic positions lowers the APB formation energy by 2 – 19% depending on the APB plane under consideration. The relaxation has the most pronounced effect on the {110} APB’s, while the formation energy of the {111} APB’s is least affected. In addition, a comparison of the effect of relaxation for the same APB’s in GaP and in GaAs revealed that the relative reduction of the formation energy is systematically higher in GaP. In the following, we discuss results that include relaxation of atomic positions.

The results for APB formation energy in GaP as a function of the APB spacing are shown in Figure 3b. The magnitude of the APB formation energy in GaP is, on average, about 20% higher than that in GaAs. Both the {110} and {111} APB’s in GaP feature trends similar to GaAs, namely an increase of the formation energy with increasing APB spacing.

The trend of increasing {110} formation energy with increasing the APB separation is likely due to the electrostatic interaction between the individual wrong bonds in different APB planes. Since the {110} APB’s are stoichiometric, the charge transfer takes place *within* the APB plane keeping it neutral on a large scale. However, when two APB’s are in close proximity, the III-III bonds in one plane face V-V bonds in the other plane as shown in Figure 2a. Owing to the fact that the III-III and V-V bonds are oppositely charged as a result of compensation, this charge distribution creates an attractive potential and lowers the formation energy. This effect is noticeable when the distance between APB’s is comparable to the intra-plane spacing between the wrong bonds. As a result, the formation energy of the {110} APB’s as a function of the APB spacing converges relatively fast as shown in Figure 3. For the distant {110} APB’s in GaAs, the wrong bond energy *E*_wb_ was calculated to be 0.31 eV (Table 1), which is close to the energy of 0.3 eV per bond estimated for an antisite pair (Ga)_As_ + (As)_Ga_ [15].

The trend towards increasing {111} APB formation energy with increasing APB spacing was previously discussed in Refs. [16, 18] in light of the energy gain due to the charge transfer across the APB from donor to acceptor states, which is hindered by a zigzag potential cased by the ionization of these states. Increasing the APB spacing lowers the magnitude of the charge transfered between two APB’s, which results in an increase of the APB formation energy (Figure 3). The critical separation between non-stoichiometric APB’s at which the APB electronic structure turns into the metallic state can be estimated as [16]

(2) $$L_{c}\;=\;\frac{2E_{g}{ɛ}_{r}{ɛ}_{0}}{\sigma }$$

where *E*_g_ is the energy gap of a semiconductor, ε_r_ is the static dielectric constant, ε_0_ is the permittivity of free space, and σ is the APB areal charge density associated with wrong bonds under assumption of the full compensation. The substitution of *E*_g_ = 1.5 eV and ε_r_ = 13 for GaAs, combined with the substitution of *E*_g_ = 2.3 eV and ε_r_ = 11 for GaP yields *L*_c_ ≈6 – 7 Å for {111} APB’s. In calculations, we observe the metallic transition for {111} APB’s at the separation of 6 – 10 Å. In order to explore the saturation of the formation energy with DFT, a relatively large (about 100 atoms) supercell is necessary to meet the condition *L* ≫*L*_c_, which is referred to as distant APB’s. The results of the calculations gathered in Table 1 suggest that the wrong bond energy for distant {111} APB’s is almost by a factor of two larger than the corresponding value for the {110} APB, which yields a magnitude of about 0.3 eV for the compensation energy per bond.

It is worth noting that the difference in the wrong bond energy associated with the {110} and {111} APB’s washes out completely for APB’s with the least possible separation, which is about 3 – 4 Å. In this case *E*_wb_ ≈ 0.26 eV and is independent of the APB type for both GaAs and GaP. This is because full compensation can be achieved at a small separation and a similar lattice of wrong bonds is formed, which results in a similar Madelung contribution to the total energy. Under these circumstances, the lowest energy has the {111} APB due to the lowest wrong bond density (Figure 3).

There is still an open question as to whether the tetragonal distortion caused by a lattice mismatch between the epilayer and the substrate can cause any changes to the trends previously discussed. The lattice mismatch between GaP and Si is small (about 0.4% at room temperature). When this effect is included in the calculations of the {110} APB’s in GaP, it has a marginal effect on the APB formation energy. The difference between specific formation energies of the strained and non-strained structures is 0.4 meV/Å^2^, which is at the limit of accuracy of the calculations. We did not perform this calculations for GaAs/Ge, as a similar result was expected owing to an even smaller value of the lattice mismatch between Ge and GaAs.

The results of the study demonstrate that the APB growth and annihilation in the {111} plane is energetically highly unfavorable. The specific formation energy of the distant {111} APB’s is by a factor of 1.5 larger than for {110} APB’s (Table 1). Next, an alternative mechanism for APB annihilation that involves APB kinking in the {112} or {113} plane, as illustrated on Figure 1(c), is considered. Ultimately, the energetic characteristics of these APB’s are compared with the {111} and {110} APB’s.

In contrast to the {111} APB that consists of only single type of wrong bonds, the {113} APB has one extra wrong bond per each pair of III-III and V-V bonds and, therefore, is partly stoichiometric. The excess stoichiometry in this case is only $\frac{1}{3}$, as compared to 1 for the {111} APB. Therefore, even for the distant APB’s, a partial intra-plane compensation is expected to take place that should lower the wrong bond energy.

The results for the formation energy of the {113} APB’s as a function of their separation are shown in Figure 3. This dependence is qualitatively different from that featured by the {110} and {111} APB’s. The formation energy initially drops with increasing of spacing between the {113} APB’s. Analysis of the electronic structure in both GaAs and GaP shows that the high initial formation energy of the {113} APB’s is associated with a metallic electronic structure. The metallic character may be attributed to a repulsive potential originating from wrong bond ordering in the {111} plane similar to that discussed above (see also discussion of the zig-zag potential in Ref. [16]). Further increasing of the APB spacing results in the emergence of an energy gap between occupied and unoccupied states with subsequent decreasing of the formation energy, as shown in Figure 3. The wrong bond energies of distant {113} APB’s are listed in Table 1. As expected, these values turn out to be only slightly higher than those for {110} APB’s. However, the high wrong bond density inherent to the {113} APB negates this effect and increases the formation energy to 39 and 46 meV/Å^2^ in GaAs and GaP, respectively.

**Figure 4.**
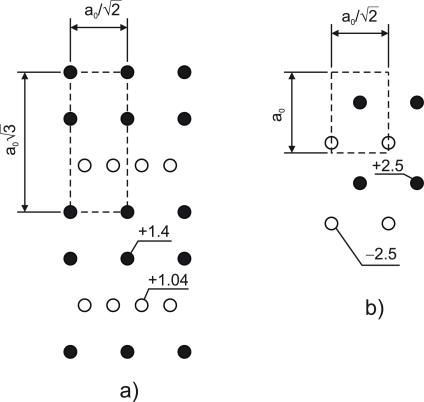
Lattice of charges in the plane of the {112} APB (a) and the {110} APB (b) that forms as a result of the charge transfer *q* between wrong bonds. The open and filled circles correspond to the positively charged V-V bonds and negatively charged III-III bonds, respectively. The primitive cell is shown by dashed lines with dimensions given in terms of the equilibrium lattice constant *a*_0_. The Madelung (Coulomb) potential due to this distribution of charges is indicated in the units of 4πε_r_ε_0_*a*_0_/*q*.

Figure 3 shows the specific formation energy of the {112} APB’s in GaAs and in GaP as a function of the APB spacing. The distant APB’s posses formation energies very close to the {113} APB (Table 1) despite the fact that the {112} APB is stoichiometric. The reason for that is an unfavorable arrangement of the charge states, as illustrated in Figure 4a, that emerges as a result of compensation. The potential experienced by a hole created at V-V bonds turns out to be positive. This implies that the ionized donor states associated with the V-V bonds will shift down in energy, thereby reducing the energy gap. Indeed, DFT calculation reveal the shrinkage of the energy gap in the case of distant {112} APB’s. This phenomenon is not observed in the {110} APB’s, since the potential experienced by electrons and holes localized at the wrong bonds has the opposite sign (Figure 4b).

Ultimately, the energetic characteristics of the {112} and {113} APB’s are so close to each other that it is hard to give preference to one or another. Although the formation energy of the {112} and {113} APB’s is considerably higher than that for the {110} APB’s, its value appears *below* the corresponding formation energy of {111} APB’s (Table 1).

## 4. Conclusions

Investigations of energetic and electronic characteristics of the {110}, {112}, {113} and {111} antiphase boundaries (APB’s) were performed using the density functional theory. Large-size supercells used in the calculations allowed us to assess the asymptotic formation energy corresponding to the distant APB’s. The formation energy of APB’s in GaP is about 20% higher than that in GaAs. Relaxation of atomic positions was performed for all structures investigated and leads to a drop in the APB formation energy of up to 19%. From all the APB’s considered, the {111} APB has the highest formation energy, while the {110} APB has the lowest energy. Both {112} and {113} APB’s have an intermediate value of the formation energy that makes these planes good candidates for the APB kinking and annihilation.

## Figures and Tables

**Table 1.** Structural and energetic characteristics of the distant APB’s in GaAs and GaP.

APB plane	W.B. density ( $\times \;a_{0}^{2}$)	Excess stoichiometry	γ(meV/Å^2^)		*E*_wb_ (eV)	
			GaAs	GaP	GaAs	GaP
{110}	$2\sqrt{2}\;\approx \;2.8$	0	28	34^1^	0.31	0.35
{111}	$4/\sqrt{3}\;\approx \;2.3$	1	43	53	0.59	0.67
{113}	$12/\sqrt{11}\;\approx \;3.6$	1/3	39	46	0.34	0.37
{112}	$8/\sqrt{6}\;\approx \;3.3$	0	39	48	0.37	0.43

1 $\gamma_{\left\{110\right\}}^{\text{GaP}/\text{Si}}\;=\;33.6\;{\text{meV}/\text{Å}}^{2}$
